# Short-term impact of preinjury antithrombotic therapy on outcomes in older trauma patients

**DOI:** 10.1007/s00068-026-03115-6

**Published:** 2026-02-23

**Authors:** Samantha Scharringa, Pieta Krijnen, Sven Meylaerts, Jephta van den Bremer, Pieter van de Linde, Jan Siert Reinders, Klaas Hartholt, Inger B. Schipper

**Affiliations:** 1 Department of Surgery, Leiden University Medical Center, Albinusdreef 2, Leiden, South-Holland 2333 ZG The Netherlands; 2 Acute Care Network West-Netherlands, Leiden, The Netherlands; 3 Department of Surgery, Haaglanden Medical Center, The Hague, The Netherlands; 4 Department of Surgery, Alrijne Hospital, Leiderdorp, The Netherlands; 5 Department of Surgery, Haga Hospital, The Hague, The Netherlands; 6 Department of Surgery, Groene Hart Hospital, Gouda, The Netherlands; 7 Department of Surgery-Traumatology, Reinier de Graaf Hospital, Delft, The Netherlands

**Keywords:** Elderly, Trauma, Antithrombotic therapy, Anticoagulant, Antiplatelet, Outcome

## Abstract

**Purpose:**

Preinjury use of antithrombotic medication (PAM) may increase bleeding risk and adverse outcomes in older trauma patients. PAM is not routinely recorded in the Dutch trauma registry, leaving its impact on outcomes unclear. This study describes PAM among older trauma patients and assesses its association with injury patterns and patient outcomes, thereby informing whether routine documentation in trauma registries would be useful.

**Methods:**

Data from trauma patients aged ≥ 65 years admitted to hospitals within the West-Netherlands trauma region in June-September 2024 were obtained from the regional trauma registry. PAM was additionally documented during this period. Poor outcome was defined as in-hospital mortality and Glasgow Outcome Scale (GOS) score ≤ 3 at hospital discharge. Associations between PAM and outcomes were analyzed using multivariable logistic regression.

**Results:**

Of 1,112 patients, 50% used antithrombotic medication, most commonly platelet aggregation inhibitors (47%) or direct oral anticoagulants (37%). Users were older, more often male, had more comorbidities, and more frequently sustained low-energy falls and minor head injuries. PAM was not associated with higher ISS (median 9 in both groups, *p* = 0.80), more intracranial hemorrhages (74% vs. 71%, *p* = 0.85), increased mortality (adjusted odds ratio [OR], 0.79; 95% Confidence Interval [CI], 0.41–1.53), or poor GOS at discharge (OR, 1.06; 95% CI, 0.79–1.43).

**Conclusion:**

PAM is common among older trauma patients. In this exploratory study, PAM did not seem to be associated with more severe injuries or poorer short-term outcomes. To evaluate the in-depth and long-term effects structured documentation of PAM in trauma registries is advocated.

**Supplementary Information:**

The online version contains supplementary material available at 10.1007/s00068-026-03115-6.

## Background

More than 40% of all trauma patients admitted to Dutch hospitals are aged over 70 years [1]. Older trauma patients often present with pre-existing conditions requiring antiplatelet and/or anticoagulant (antithrombotic) therapy, such as cardiovascular disease [2]. In the Netherlands, approximately two million individuals are prescribed antithrombotic therapy, of which 72% are aged over 65 years, and this number has been steadily rising since 2018 [3].

In the trauma setting, awareness of preinjury antithrombotic medication use is crucial, as it may exacerbate bleeding. Acute reversal or mitigation of effects of antithrombotic medication can be critical yet challenging due to patient factors influencing drug half-life and hospital variability in the availability of specific antidotes. Several studies have reported an association of preinjury antithrombotic therapy with adverse outcomes in elderly trauma patients, reflected by an increase in frequency and severity of bleeding complications [4], higher mortality [4–8], and longer hospital length of stay [6]. On the other hand it was reported that elderly patients on preinjury antithrombotic therapy with recurrent falls were not necessarily more likely to sustain a bleeding injury. Patients with a bleeding injury did have a higher risk of mortality [9]. Evidence regarding the risks of preinjury use of different anticoagulant types in trauma patients remains inconsistent. Several studies [7, 10–12] found that anticoagulant use, but not antiplatelet medication, was associated with an increased risk of mortality and hospital length of stay, while others found that specific drug classes have different effects [6, 13, 14].

The Netherlands has one of the most mature trauma systems in Europe, supported by a comprehensive national trauma registry [15]. This registry plays a vital role in evaluating trauma care and informing the development of evidence-based guidelines. Preinjury antithrombotic medication use is not actively incorporated into the prehospital trauma triage protocol used by Dutch ambulance services [16], nor is it recorded in the Dutch trauma registry. This omission is concerning, as standard prehospital triage protocols have been shown to be insufficient in identifying intracranial hemorrhages [17]. The unavailability of these data obstructs systematic monitoring of antithrombotic medication exposure in Dutch trauma patients and limits the ability to develop evidence-based guidelines to enhance prehospital management and triage strategies for this high-risk group.

Acknowledging that there is no consensus on the effects of preinjury antithrombotic medication use on older trauma patients and that no data on this topic are currently available for the Dutch trauma population, this exploratory study aims to characterize the preinjury use of antithrombotic medication among older trauma patients and to investigate the association between antithrombotic medication use, injury patterns, and patient outcomes. The findings may help determine whether routine registration of antithrombotic medication should be implemented in the Dutch trauma registry and incorporated into the prehospital trauma triage protocol.

## Methods

### Study design and population

This retrospective cohort study was conducted in the West Netherlands trauma region in two level-1 trauma centers and five level-2/3 trauma hospitals. Data of trauma patients aged ≥ 65 years, admitted within 48 h after trauma between June and September 2024, were extracted from the regional trauma registry. For the purpose of this study, information on preinjury antithrombotic medication use was additionally registered during the study period. Patients transferred to or from another hospital were excluded to avoid duplicate records.

This study does not fall under the Dutch Medical Research Involving Human Subjects Act (WMO) and was therefore exempt from review by a Medical Ethics Committee. Informed consent was not required as the study involved no direct patient contact and was based on retrospectively collected data from the regional trauma registry.

### Study parameters

The following variables were extracted from the trauma registry: patient characteristics, preinjury antithrombotic medication use and type, injury characteristics, prehospital level of consciousness, hospital admission outcomes (CT scan made (yes/no), length of stay, intensive-/high-/medium care unit [ICU/HCU/MCU] admission), and patient outcomes (poor functional outcome at discharge, defined as Glasgow Outcome Scale (GOS) score ≤ 3 [18] and in-hospital mortality). Age was categorized into “65–74 years”, “75–84 years”, and “≥85 years”. Comorbidity was classified using the American Society of Anesthesiologist’s Physical Status Classification System (ASA Score) [19]. Cause of injury was dichotomized into “low-energy fall” and “other”. Injuries were considered severe for Abbreviated Injury Scale (AIS) scores with the last digit ≥ 3 and multitrauma was defined as Injury Severity Score (ISS) ≥ 16. Prehospital level of consciousness was categorized into severely compromised (Glasgow Coma Scale (GCS) 3–8), moderate (GCS 9–12), and mild (GCS 13–15) according to the Advanced Trauma Life Support (ATLS) classification [20]. Intracranial hemorrhages were subdivided into the following diagnoses: epidural/extradural hematoma, intracerebral hematoma (including diffuse axonal injury and intraventricular hemorrhage), subdural hematoma, intraventricular hemorrhage, subarachnoid hemorrhage, and subpial hemorrhage (see the Appendix for the list of corresponding Abbreviated Injury Scale (AIS) codes). In the trauma registry, the severity of the intracranial hemorrhages is not per se based on the first CT scan made upon presentation at the emergency department. Rather, it is determined based on the most severe finding on the CT scan up to three months after initial injury presentation. In this study, short-term outcomes were defined as outcomes occurring during hospital admission, in accordance with the standard three-month follow-up period recorded in the Dutch trauma registry.

### Statistical analysis

Statistical analysis was performed using IBM SPSS version 31. Statistical significance was set at *p* < 0.05. Categorical data are presented as number (percentage) and continuous data as median (range or interquartile range) or mean (standard deviation (SD)). Antithrombotic medication use (yes or no) was compared using the Student t-test, depending on statistical distribution or Mann-Whitney test for continuous data and the Pearson’s chi-squared test or Fisher’s exact test for categorical data. Differences among the different subtypes of antithrombotic medication were evaluated using the ANOVA or Kruskal-Wallis test for continuous data, and Pearson’s chi-squared test for categorical data.

To evaluate the effect of antithrombotic medication use on in-hospital mortality and poor functional outcome, multivariable logistic regression analysis was conducted, with adjustment for confounding. Potential confounders were identified as patient and injury characteristics having a univariable association (*p* < 0.05) with preinjury antithrombotic drug use.

## Results

### Preinjury use of antithrombotic medication

**Fig. 1:**
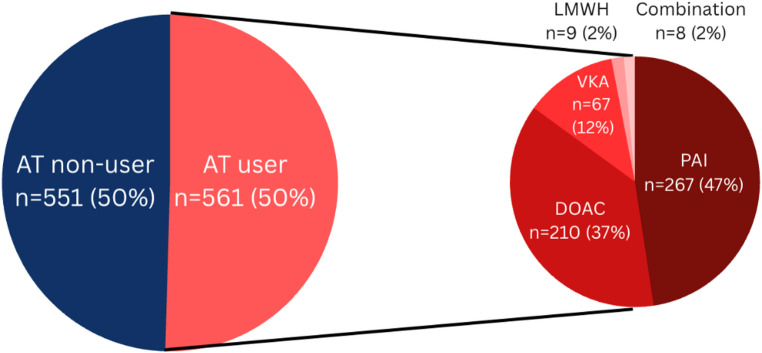
Patient distribution. AT – Antithrombotic drug; PAI – Platelet Aggregation Inhibitor; VKA – Vitamin K antagonist; LMWH – Low Molecular Weight Heparin; DOAC – Direct Oral Anticoagulant

A total of 1,112 elderly trauma patients were registered during the study period, of whom 561 (50%) used preinjury antithrombotic medication (Fig. 1). Platelet aggregation inhibitors were used most frequent (PAIs) (47%), followed by direct oral anticoagulants (DOACs) (37%), vitamin K antagonists (VKAs) (12%) and low molecular weight heparin (LMWH) (2%). Eight patients used a combination of antithrombotic medication.

Antithrombotic medication users were more often male, older, and had more severe comorbidities (Table 1). They also more frequently suffered low-energy falls compared to non-users (82% vs. 74%, respectively, *p* < 0.001). The rate of multitrauma was 6% in both groups. Injury severity score (ISS), prehospital GCS, trauma center level distribution, and the distribution of severe injuries across body regions was not statistically different between antithrombotic medication users and non-users. Severe injuries to the neck, abdomen, and upper extremities were not analyzed due to small numbers.

The incidence of all head injuries (AIS ≥ 1, including isolated concussion) was higher in patients using preinjury antithrombotic medication compared to non-users (32% vs. 26%, respectively, *p* = 0.02), while the incidence of moderate to severe head injuries (AIS ≥ 2) was similar between groups (14% in non-users vs. 15% in users, *p* = 0.52). The incidence of intracranial hemorrhage (74% vs. 71%, *p* = 0.76) and the severity of intracranial hemorrhage (median [range] AIS 3 [2–5] vs. 3 [2–5], *p* = 1.00) did not differ between non-users and users. Subdural hematoma (34% vs. 43%, *p* = 0.36) and subarachnoid hemorrhage (34% vs. 30%, *p* = 0.87) were the most common types of isolated intracranial hemorrhage in both groups. The prevalence of specific types of isolated intracranial hemorrhage of multiple intracranial hemorrhages was not statistically different between antithrombotic medication users and non-users (Table 1).

**Table 1:** Patient and injury characteristics by preinjury antithrombotic drug use

		No antithrombotic drug use (*n* = 551)	Antithrombotic drug use (*n* = 561)	*p*-value
Male, *n* (%)		174 (32)	225 (40)	0.003
Age, mean ± SD		79 ± 8.4	82 ± 7.7	**< 0.001**
Age categories, n (%)				**< 0.001**
65–74 years		187 (34)	111 (20)	
75–84 years		212 (38)	234 (42)	
≥ 85 years		152 (28)	216 (38)	
Comorbidity, n (%)				**< 0.001**
No systemic disease (ASA 1)		46 (8)	0 (0)	
Mild systemic disease (ASA 2)		264 (48)	121 (22)	
Severe systemic disease (ASA 3)		222 (40)	398 (71)	
Very severe systemic disease (ASA 4)		18 (3)	41 (7)	
Cause of injury, n (%)				**< 0.001**
Low-energy fall		405 (74)	461 (82)	
Other		146 (26)	100 (18)	
ISS, median (IQR)		9 (5–9)	9 (5–10)	0.80
Multitrauma (ISS ≥ 16), n (%)		31 (6)	32 (6)	1.00
Trauma center level				0.12
Level-1, n (%)		307 (56)	286 (51)	
Level-2/3, n (%)		244 (44)	275 (49)	
Prehospital GCS				0.23
Severe (3–8)		8 (1)	3 (1)	
Moderate (9–12)		4 (1)	6 (1)	
Mild (13–15)		539 (98)	552 (98)	
Severe Injury (AIS ≥ 3) per body region#, n (%)				
Thorax		45 (8)	37 (7)	0.32
Spinal		14 (3)	8 (1)	0.18
Lower extremities		280 (51)	277 (49)	0.21
Head injury (AIS ≥ 1)		141 (26)	178 (32)	**0.02**
Head injury (AIS ≥ 2)		76 (14)	85 (15)	0.52
Intracranial hemorrhage		56/76 (74)	60/85 (71)	0.85
Intracerebral hematoma or diffuse axonal injury only, n (%)		2/56 (4)	1/60 (2)	0.62
Epidural hematoma only, n (%)		0/56 (0)	1/60 (2)	1.00
Subdural hematoma only, n (%)		19/56 (34)	26/60 (43)	0.36
Subarachnoid hemorrhage only, n (%)		19/56 (34)	18/60 (30)	0.87
Multiple hemorrhages, n (%)		16/56 (28)	14/60 (23)	0.71
Severity (AIS) of intracranial hemorrhage, median (range)		3 (2–5)	3 (2–5)	1.00

ASA –American Society of Anesthesiologist’s Physical Status Classification System; ISS – Injury Severity Score; AIS – Abbreviated Injury Scale; GCS – Glasgow Coma Scale; SD – Standard Deviation; IQR – Interquartile Range

# Severe injuries to the abdomen and upper extremities were not analyzed due to small numbers

### Type of preinjury antithrombotics

Since only 9 patients used preinjury low-molecular weight heparin (LMWH), this group was not further analyzed. PAI users were statistically younger compared to DOAC and VKA users (Table 2). No statistically significant differences were observed for gender and comorbidity severity across antithrombotic drug types. Frequency of low-energy falls, trauma center level distribution, prehospital GCS, ISS, and rate of multitrauma also did not differ significantly between these groups. Compared to PAI and VKA users, DOAC users more often had severe injuries to the lower extremities (43% vs. 56% vs. 49%, *p* = 0.03).

The incidence of any head injury (AIS ≥ 1, including isolated concussion) was the highest among PAI users (38% vs. 26% vs. 30%, *p* = 0.02), but no statistically significant difference in the incidence of moderate to severe head injuries (AIS ≥ 2) was found between antithrombotic types (18% vs. 14% vs. 8%, *p* = 0.11). The incidence of intracranial hemorrhage was higher among PAI and DOAC users than among VKA users (77% vs. 71% vs. 20%, *p* = 0.02). No statistically significant differences in the distribution of isolated intracranial hemorrhage diagnoses, the prevalence of multiple intracranial hemorrhages, and the severity of intracranial hemorrhages between the antithrombotic medication groups were found.

**Table 2:** Patient and injury characteristics by type of preinjury antithrombotic medication

		PAI (*n* = 267)	DOAC (*n* = 217)	VKA (*n* = 67)	*p*-value
Male, *n* (%)		106 (40)	85 (39)	29 (43)	0.83
Age, mean ± SD		81 ± 8.1	83 ± 7.2	83 ± 7.1	**< 0.001**
Age categories, n (%)					**< 0.001**
65–74 years		72 (27)	29 (13)	7 (10)	
75–84 years		109 (41)	89 (41)	32 (48)	
≥ 85 years		86 (32)	99 (46)	28 (42)	
Comorbidity, n (%)					0.06
No systemic disease (ASA 1)		0 (0)	0 (0)	0 (0)	
Mild systemic disease (ASA 2)		69 (26)	39 (18)	9 (13)	
Severe systemic disease (ASA 3)		180 (68)	157 (72)	55 (82)	
Very severe systemic disease (ASA 4)		17 (6)	21 (10)	3 (5)	
Cause of injury, n (%)					0.20
Low-energy fall		211 (79)	185 (85)	56 (84)	
Other		56 (21)	32 (15)	11 (16)	
ISS, median (IQR)		9 (5–10)	9 (5–9)	9 (5–9)	0.76
Multitrauma, n (%)		12 (5)	16 (7)	3 (5)	0.40
Trauma center level					0.10
Level-1, n (%)		127 (48)	117 (54)	41 (61)	
Level-2/3, n (%)		140 (52)	100 (46)	26 (39)	
Prehospital GCS					0.75
Severe (3–8)		2 (1)	1 (1)	0 (0)	
Moderate (9–12)		2 (1)	4 (2)	0 (0)	
Mild (13–15)		263 (98)	212 (97)	67 (100)	
Concomitant severe Injury (AIS ≥ 3) in other body regions#, n (%)					
Thorax		17 (6)	11 (5)	9 (13)	0.07
Spinal		4 (2)	4 (2)	0 (0)	0.80
Lower extremities		116 (43)	121 (56)	33 (49)	**0.03**
Head injury (AIS ≥ 1)		100 (38)	56 (26)	20 (30)	**0.02**
Head injury (AIS ≥ 2)		47 (18)	31 (14)	5 (8)	0.11
Intracranial hemorrhage		36/47 (77)	22/31 (71)	1/5 (20)	**0.02**
Intracerebral hematoma or diffuse axonal injury, n (%)		1/36 (3)	0/22 (0)	0/1 (0)	1.00
Epidural hematoma, n (%)		1/36 (3)	0/22 (0)	0/1 (0)	1.00
Subdural hematoma, n (%)		14/36 (39)	11/22 (50)	0/1 (0)	0.14
Subarachnoid hemorrhage, n (%)		12/36 (33)	5/22 (23)	1/1 (100)	0.37
Multiple hemorrhages, n (%)		8/36 (22)	6/22 (27)	0/1 (0)	0.48
Severity (AIS) of intracranial hemorrhage (median [range])		3 (2–5)	3 (2–5)	2 (2–2)	0.85

PAI – Platelet Aggregation Inhibitor; VKA – Vitamin K antagonist; DOAC – Direct Oral Anticoagulant; ASA – American Society of Anesthesiologist’s Physical Status Classification System; ISS – Injury Severity Score; GCS – Glasgow Coma Scale; AIS – Abbreviated Injury Scale

# Severe injuries to the abdomen and upper extremities were not analyzed due to small numbers

### Hospital admission and patient outcomes

Antithrombotic medication users underwent CT scans more frequently than non-users (48% vs. 40%, *p* = 0.01) (Table 3). Among all patients with head injuries (AIS ≥ 1), however, the proportion undergoing CT imaging was similar between the two groups. Antithrombotic medication users also had a longer hospital stay (median [IQR] 7 [4–9] vs. 6 [3–8] days, *p* < 0.001), while the number of patients admitted to the intensive-/high-/medium care unit (ICU/HCU/MCU) was comparable between groups.

In-hospital mortality did not differ between users and non-users (5% vs. 4%, *p* = 0.26). Also, no difference in in-hospital mortality was found after adjusting for confounders (adjusted odds ratio [OR], 0.79; 95% Confidence Interval [CI], 0.41–1.53; *p* = 0.49). In the univariable analysis, preinjury antithrombotic medication use was associated with a higher risk of having poor GOS at discharge (68% vs. 57%, *p* < 0.001; unadjusted OR, 1.64; 95% CI, 1.28–2.10; *p* < 0.001). After adjustment for confounders, this association was no longer statistically significant (OR 1.06, 95% CI 0.79–1.43; *p* = 0.69).

When comparing different types of the most commonly used antithrombotic medication (Table 4), no statistically significant differences were observed in frequency of CT scans made (including among those with head injuries), length of stay, admission to the ICU/HCU/MCU, GOS at discharge, or in-hospital mortality.

**Table 3:** Hospital admission and patient outcomes by preinjury antithrombotic drug use

		No antithrombotic drug use (*n* = 551)	Antithrombotic drug use (*n* = 561)	*p*-value	Univariate	Multivariate**
CT scan ordered, *n* (%)		221 (40)	269 (48)	0.01		
In patients with head injuries (AIS ≥ 1)		131/141(93)	169/178 (95)	0.48		
LOS, median (IQR)		6 (3–8)	7 (4–9)	**< 0.001**		
ICU/HCU/MCU admission, n (%)		28 (5)	29 (5)	0.95		
In-hospital mortality, n (%)		20 (4)	28 (5)	0.26	1.40 (0.78–2.51)	0.79 (0.41–1.53)
Poor functional outcome at discharge^1^, n (%)		312 (57)	383 (68)	**< 0.001**	1.64 (1.28–2.10)	1.06 (0.79–1.43)

LOS – Length of Stay; ICU – Intensive Care Unit; HCU – High Care Unit; MCU – Medium Care Unit; IQR – interquartile range

^1^Poor functional outcome was defined as Glasgow Outcome Scale score ≤ 3

**Adjusted for gender, age, comorbidity (based on the American Society of Anesthesiologist’s Physical Status Classification System), cause of injury (low-energy fall or other trauma mechanisms), head injuries (AIS ≥ 1)

**Table 4:** Hospital admission and patient outcomes by type of preinjury antithrombotic medication

		PAI (*n* = 267)	DOAC (*n* = 217)	VKA (*n* = 67)	*p*-value
CT scan ordered, *n* (%)		131 (49)	102 (47)	33 (49)	0.89
In patients with head injuries (AIS ≥ 1)		93/100 (93)	54/56 (96)	20/20 (100)	0.54
LOS, median (range)/(IQR)		6 (4–9)	7 (5–10)	7 (5–10)	0.21
ICU/HCU/MCU admission, n (%)		12 (5)	11 (5)	5 (8)	0.56
In-hospital mortality, n (%)		10 (4)	14 (6)	4 (6)	0.22
Poor functional outcome at discharge^1^, n (%)		169 (63)	156 (72)	50 (75)	0.06

PAI – Platelet Aggregation Inhibitor; VKA – Vitamin K antagonist; DOAC – Direct Oral Anticoagulant; LOS – Length of Stay; ICU – Intensive Care Unit; HCU – High Care Unit; MCU – Medium Care Unit; IQR – interquartile range

^1^Poor functional outcome was defined as Glasgow Outcome Scale score ≤ 3

## Discussion

More than half of the elderly trauma patients presenting to the emergency department used preinjury antithrombotic medication, most commonly PAIs or DOACs. Compared to non-users, antithrombotic medication users were generally older, more often male, had more often severe comorbidities and a low-energy fall as trauma mechanism. Injury patterns and severity of injury sustained by antithrombotic medication users and non-users were comparable in all trauma patients as well as in the subgroup of patients with head injury. Preinjury antithrombotic medication use was not a risk factor for in-hospital mortality or poor functional outcome at discharge after adjusting for confounders. Among the three most used antithrombotic medication types (PAI, VKA, DOAC), PAI users had the highest rate of head injuries, while VKA users had the lowest rate of intracranial hemorrhage, although this result should be interpreted with caution given the small number of patients using VKAs.

In this study examining the impact of preinjury antithrombotic medication use on outcomes in Dutch elderly trauma patients, preinjury antithrombotic therapy was not associated with a higher incidence of intracranial hemorrhage or poor clinical outcomes. These findings contrast with several earlier studies that report worse outcomes among elderly trauma patients receiving antithrombotic therapy [4–8, 11, 12, 21, 22]. The differences maybe be explained by differences in study population, case mix, and outcome measures. Unlike most previous studies that focused exclusively on patients with traumatic brain injury (TBI) [4, 7, 12, 13, 21–24] or fall-related trauma [6, 7, 9, 13, 23, 25], this regional cohort study included all geriatric trauma admissions, resulting in a broader and less selectively injured population, despite ground-level falls remaining the most common injury mechanism. Furthermore, this study assessed in-hospital mortality and Glasgow Outcome Scale scores at discharge, whereas other studies used alternative endpoints such as 30-day mortality [5, 14, 21, 22, 24] or radiologic progression of TBI on follow-up CT imaging [4, 26–29]. Finally, whereas many earlier studies evaluated specific antithrombotic agents [6, 11, 13, 14], this analysis examined outcomes across antiplatelet (PAI) and anticoagulant (VKA and DOAC) users in comparison with non-users. However, subgroup analyses were limited by sample size, particularly for patients with specific injury patterns such as intracranial hemorrhage. For instance, increased bleeding risk associated with VKAs – which is predominantly reported in TBI-restricted cohorts [4, 21] – could not be reliably evaluated in the present study due to the limited number of VKA users with head injures, which may partly explain the absence of such associations in this broader geriatric trauma population.

Alternatively, the findings of the present study are in line with those of Rønning et al. (2021) [24], who studied a mixed cohort of patients with traumatic brain injury (TBI) and other injuries, including multitrauma. They also found that antithrombotic monotherapy was not associated with an increased risk of mortality, supporting the plausibility of our results. Additionally, when focusing specifically on patients with head injuries, comparable findings were reported by Fakhry et al. (2021) [23] and Nishijima et al. (2018) [30], who studied elderly patients with TBI and found no association between preinjury antithrombotic use and intracranial hemorrhage.

In this cohort, approximately one-third of both antithrombotic medication users and non-users sustained head injuries, and among these patients, the incidence of intracranial hemorrhage was similar between users of antithrombotics and non-users. However, these findings should be interpreted with caution. The relatively short study period resulted in limited sample sizes, reducing statistical power to detect differences in rare but clinically important outcomes such as (delayed) intracranial hemorrhage, and limits generalizability by increasing the risk of seasonal and selection bias. Accordingly, the absence of significant associations in the present study should not be interpreted as evidence of safety or equivalence of antithrombotic therapy in patients with moderate to severe TBI. Thus, larger population-based research specifically focusing on TBI patients with sufficient statistical power for subgroup analyses is needed to confirm these findings.

Within the context of this cohort, the findings do not support inclusion of preinjury antithrombotic use as a stand-alone prehospital triage criterion. However, given the limited number of patients with moderate to severe TBI, these findings should not be interpreted as definitive evidence against a role for antithrombotic use in triage decisions for high-risk groups. This interpretation is supported by findings from a systematic review [31] of United States’ Field Triage Guidelines, which reported that anticoagulant use was not a major predictor of serious injury. Accordingly, in the most recent revision of these guidelines [32], anticoagulant use is not considered under “EMS judgement” rather than as a stand-alone criterion for transport to a higher-level trauma center. This approach appears justified, as EMS judgement – particularly for predicting intracranial hemorrhage in head-injured older adults – has been shown to perform well in practice [33].

### Limitations

This study is not without limitations. First, the limited three-month inclusion period resulted in a relatively small sample size for subgroups of patients with uncommonly used types of antithrombotic medication (VKA, LMWH), and with combination therapies. Consequently, only the three most frequently used medication types (PAI, DOAC, VKA) were analyzed, where a cautious interpretation of the results, especially for VKA users, is warranted. Additionally, only 29% of the study population sustained a head injury (and only 15% a moderate to severe head injury), which limits the statistical power for finding differences in injury patterns and outcomes for patients with TBI. Such a short inclusion period may also introduce seasonal variation and selection bias, potentially limiting representativeness of injury mechanisms and severity. Second, data on the International Normalized Ratio (INR), a measure of coagulation status, could not be included in the analyses due to incomplete registration, resulting in a high proportion of missing values. Third, as this study is dependent on variables routinely recorded in the Dutch trauma registry, information on timing of last antithrombotic medication intake and medication adherence was unavailable. These unmeasured variables may have influenced bleeding risk and outcomes, resulting in residual confounding. Fourth, data on acute (neuro)surgical interventions were not analyzed due to the low number of patients undergoing emergency surgery (1.1%). Fifth, data on the use of reversal therapy were unavailable, preventing analysis of which patients received reversal agents and whether this influenced outcomes. Lastly, the Dutch trauma registry only tracks outcomes for three months, preventing the assessment of long-term morbidity and mortality.

## Conclusion

Approximately half of the elderly trauma patients presenting to the emergency department used preinjury antithrombotic medication, most commonly PAIs or DOACs. In this regional cohort, preinjury antithrombotic use was not associated with increased injury severity or short-term poor patient outcomes. However, given the limitations of this exploratory study described above, these findings should be interpreted with caution. Routine registration of preinjury antithrombotic medication use in trauma registries is therefore strongly recommended. Registration would support large-scale, longitudinal, and national and international comparative studies; facilitate detailed subgroup analyses, including patients with TBI, users of less common antithrombotic agents, and patients receiving combination therapy; enable identification of rare but high-risk subgroups; allow epidemiological monitoring of antithrombotic use trends; inform refinement of trauma triage and CT-imaging decision protocols; and ultimately guide evidence-based policy-making and trauma system improvement.

## Supplementary Information

Below is the link to the electronic supplementary material.

Supplementary Material 1

## Data Availability

Data from the regional trauma registry are available upon request.
