# Supplementary material for: Short-term impact of preinjury antithrombotic therapy on outcomes in older trauma patients
Source: Eur J Trauma Emerg Surg. 2026 Feb 23;52(1):57. doi: 10.1007/s00068-026-03115-6 (PMC12929266; doi:10.1007/s00068-026-03115-6)
Supplement: Supplementary file 1 — Supplementary Material 1 [file 68_2026_3115_MOESM1_ESM.docx]

#### Appendix 1. List of intracranial hemorrhages diagnoses and corresponding AIS codes

| Epidural hemorrhage | | |
| --- | --- | --- |
|  | 1404143 | Epidural or extradural NFS |
|  | 1404162 | Epidural or extradural NFS - tiny |
|  | 1404184 | Epidural or extradural NFS – small/moderate |
|  | 1404225 | Epidural or extradural NFS - large |
|  | 1406303 | Epidural or extradural NFS |
|  | 1406312 | Epidural or extradural NFS - tiny |
|  | 1406324 | Epidural or extradural NFS – small/moderate |
|  | 1406345 | Epidural or extradural NFS – bilateral small/moderate |
|  | 1406365 | Epidural or extradural NFS - large |
|  | 1406293 | Extra-axial hemorrhage NFS |
| Subdural hemorrhage | | |
|  | 1404383 | Subdural NFS |
|  | 1404402 | Subdural NFS - tiny |
|  | 1404424 | Subdural NFS – small/moderate |
|  | 1404465 | Subdural NFS - large |
|  | 1406503 | Subdural NFS |
|  | 1406513 | Subdural NFS - tiny |
|  | 1406524 | Subdural NFS – small/moderate |
|  | 1406544 | Subdural NFS – bilateral small/moderate |
|  | 1406565 | Subdural NFS - large |
|  | 1406555 | Subdural NFS – bilateral large |
| Subarachnoid hemorrhage | | |
|  | 1404662 | Subarachnoid hemorrhage |
|  | 1406932 | Subarachnoid hemorrhage NFS |
|  | 1406942 | Subarachnoid hemorrhage not associated with coma >6 hours |
|  | 1406953 | Subarachnoid hemorrhage associated with coma >6 hours |
| Subpial hemorrhage | | |
|  | 1404702 | Subpial hemorrhage |
|  | 1406962 | Subpial hemorrhage NFS |
|  | 1406972 | Subpial hemorrhage not associated with coma >6 hours |
|  | 1406983 | Subpial hemorrhage associated with coma >6 hours |
| Intracerebral hematomas | | |
|  | 1404103 | Cerebellum hematoma NFS |
|  | 1404263 | Intracerebellar incl. petechial and subcortical NFS |
|  | 1404282 | Intracerebellar - tiny |
|  | 1404304 | Intracerebellar – small |
|  | 1404345 | Intracerebellar – large |
|  | 1406284 | Diffuse axonal injury (DAI) NFS |
|  | 1406254 | DAI confined to white matter or basal ganglia |
|  | 1406275 | DAI involving corpus callosum |
|  | 1406383 | Intracerebral NFS |
|  | 1406392 | Intracerebral NFS - tiny |
|  | 1406422 | Intracerebral NFS – tiny with petechial hemorrhages |
|  | 1406432 | Intracerebral NFS – tiny with petechial hemorrhages not associated with coma >6 hours |
|  | 1406454 | Intracerebral NFS – tiny with petechial hemorrhages associated with coma >6 hours |
|  | 1406404 | Intracerebral NFS - small |
|  | 1406473 | Intracerebral NFS - small not associated with coma >6 hours |
|  | 1406494 | Intracerebral NFS - small associated with coma >6 hours |
|  | 1406465 | Intracerebral NFS – bilateral small |
|  | 1406485 | Intracerebral NFS - large |
|  | 1406415 | Intracerebral NFS – bilateral large |
|  | 1406782 | Intraventricular hemorrhage |
|  | 1406752 | Intraventricular hemorrhage not associated with coma >6 hours |
|  | 1406774 | Intraventricular hemorrhage associated with coma >6 hours |
|  | 1610074 | Diffuse axonal injury (DAI) NFS |
|  | 1610084 | Mild DAI (LOC 6-24 hours) |
|  | 1610115 | LOC >24 hours NFS |
|  | 1610125 | Moderate DAI (without brainstem signs) |
|  | 1610135 | Severe DAI (with brainstem signs) |
